# Editorial for Advances in Biostimulant Use on Horticultural Crops, First Edition

**DOI:** 10.3390/plants15081139

**Published:** 2026-04-08

**Authors:** Raphael Ofoe, Mason T. MacDonald, Lord Abbey

**Affiliations:** Department of Plant Food and Environmental Sciences, Faculty of Agriculture, Dalhousie University, Bible Hill, NS B2N 5E3, Canada

## 1. Introduction

The role of horticultural crops (fruits, vegetables, and ornamentals) is vital globally, not only for nutrition, but also for economic and cultural value. Horticultural industries are challenged by increasing populations, climate volatility, and a growing demand for sustainable, nutrient-dense horticultural crop production. The use of biostimulants has emerged not only as an agricultural input, but as a crucial strategic tool for researchers and farmers to enhance crop productivity and resilience to diverse environmental stresses.

Biostimulants are defined as any substance or microorganisms applied to plants to enhance nutrient use efficiency, abiotic stress tolerance, and/or crop quality traits, independent of their nutrient composition [1]. Unlike fertilizers that supply plants with nutrients, biostimulants function by modulating physiological, biochemical, and molecular processes in plants [2]. Horticultural crops are particularly receptive to biostimulant strategies due to their sensitivity to environmental and management stresses, their premium quality requirements, and the opportunities for targeted growth improvement.

This Special Issue brings together a timely and diverse collection of original research and reviews focused on the application of biostimulants in horticultural crops, exploring their mechanistic bases, practical efficacy, crop-specific responses, and opportunities to reduce the reliance on synthetic inputs. The response to our call for papers has been robust, yielding a collection of thirteen insightful studies that collectively advance our understanding of biostimulants. The research presented here spans the horticultural value chain, from seed priming and early establishment to postharvest quality management, and investigates a wide range of biostimulant sources and their effects on a diversity of crops. This Special Issue focuses on three thematic areas: novel and sustainable biostimulants, enhanced functional and nutritional quality, and the mitigation of abiotic stress.

### 1.1. Novel and Sustainable Biostimulants

Novel biostimulants continue to be used in horticulture in innovative ways, which is a major highlight in this Special Issue. For instance, Senthilkumar et al. [3] investigated the potential of pyroligneous acid, a by-product of pyrolysis, to improve postharvest needle retention in balsam fir. Although the direct effect on needle retention was inconsistent, the study revealed that pyroligneous acid could induce protective physiological responses, such as proline accumulation and reduced membrane injury. This represents an unforeseen benefit in balsam fir beyond the researchers’ set objective. Similarly, Varga et al. [4] demonstrated that water extracts from industrial hemp inflorescences can serve as effective biostimulants, significantly enhancing early growth, biomass, and antioxidant activity in oilseed pumpkin seedlings. This work opens avenues for valorizing agricultural by-products as novel biostimulant inputs. Other featured research included the exploration of plant extract-, fungi-, and protein hydrolysate-based biostimulants [5,6,7].

### 1.2. Enhanced Functional and Nutritional Quality

The ability of biostimulants to enhance the nutritional and functional quality of horticultural crops is another critical area of advancement. Lopes et al. [5] clearly show that foliar applications of seaweed extract (*Ecklonia maxima*) and glycine betaine can considerably regulate the accumulation of bioactive compounds and antioxidant capacity in blueberries. Importantly, these findings emphasize that the efficacy of these biostimulants is cultivar- and dose-dependent, which emphasizes the need for precise, tailored application strategies to maximize benefits. This theme of quality improvement is further supported by Lam et al. [6], who found that moderate concentrations of seaweed extract optimized growth, photosynthetic performance, and the accumulation of health-promoting phenolic compounds, such as rosmarinic acid, in Thai basil grown in controlled plant factories. In fact, most studies in this Special Issue describe results that include phytochemical changes rather than only describing growth-promoting properties. This points to an increased mechanistic interest in biostimulant use.

### 1.3. Mitigation of Abiotic Stress

Biostimulants have long been known to enhance growth characteristics, even during abiotic stress, thereby mitigating some of the deleterious stress effects. This Special Issue features six papers that specifically examine the stress-mitigating properties of biostimulants [3,8,9,10,11,12]. Featured abiotic stresses include water deficit, salinity, alkalinity, and nitrogen deficiency. Other studies, though not directly assessing stress mitigation, studied increases in antioxidant phytochemicals induced by biostimulants [4,6]. Such antioxidant increases align with a potential ability of associated biostimulants to mitigate stress.

## 2. Conclusions and Future Research

Collectively, the papers in this Special Issue illuminate several key trajectories for biostimulant use in horticulture. Firstly, they confirm the remarkable diversity of materials with biostimulant action and their potential to improve both productivity and quality. Additionally, they highlight the intricate physiological and molecular mechanisms through which these biostimulants function, moving beyond simple growth promotion to include stress modulation and secondary metabolite production. Most importantly, these papers demonstrate the critical importance of context, where the specific biostimulant, concentration, method and timing of application, and target crop are critical considerations. As we look to the future, the research compiled in this issue provides a strong foundation for continued exploration. We see a clear need for deeper mechanistic studies to fully unravel the signaling pathways and genetic networks modulated by these biostimulants. Furthermore, translating this knowledge into practical, reliable recommendations for growers remains a paramount goal. This requires not only continued discovery of new products, but also rigorous, multi-location field trials that account for the complex environmental variables of commercial production.

## Data Availability

No new data was generated for this article.
